# Etiology and prognosis of non-Kawasaki disease induced coronary aneurysms in children: a retrospective case series study

**DOI:** 10.1007/s00431-024-05666-5

**Published:** 2024-07-11

**Authors:** Yao Lin, Huiru Qi, Yanyan Liu, Haojie Wu, Yaqi Li, Lin Shi

**Affiliations:** 1 Department of Pediatric Cardiology, Children’s Hospital, Capital Institute of Pediatrics, No 2 Yabao Road, Beijing, 100020 Chaoyang District China; 2 Capital Institute of Pediatrics, Peking University Teaching Hospital, Beijing, China; 3 Chinese Academy of Medical Sciences & Peking Union Medical College, Children’s Hospital Capital Institute of Pediatrics, Beijing, China

**Keywords:** Coronary aneurysm, Kawasaki disease, Coronary artery fistula, Prognosis, Child

## Abstract

While Kawasaki disease (KD) induced coronary artery aneurysms (KD CAAs) in children are well studied, the features and prognosis of non-KD induced CAAs (non-KD CAAs) in the pediatric population are poorly documented. This case series study is to analyze the etiology and prognosis of non-KD CAAs in children and compare the characteristics of non-KD CAAs and KD CAAs. Non-KD CAA and KD CAA cases at our department from January 2022 to December 2023 were retrospectively collected. Etiologies and prognosis of non-KD CAAs were analyzed. Furthermore, demographic data, biochemical parameters and outcomes between children with Non-KD CAAs and children with KD CAAs were comparatively studied. Fifteen children with non-KD CAAs with a median age of 6 years and 117 children with KD CAAs with a median age of 2.0 years (*p* = 0.022) were included in this study. The causes of non-KD CAAs include: unknown etiologies (2 cases), coronary artery structural abnormalities (4), Takayasu arteritis (2), virus infection (2), cardiomyopathy (2), aplastic anemia with agranulocytosis (1), ANCA-associated vasculitis (1), and mucopolysaccharidosis (1). In the non-KD CAA group, there were a total of 19 CAAs with 3 being giant, 5 medium, and 11 small; 4 patients had complete CAA regression; an infant with a fistula between the right coronary artery and the coronary sinus complicated with cardiac enlargement died of heart failure. The KD group had significantly higher levels of CRP, white cells counts and ESR with zero mortality. Non-KD CAA cases had a significantly lower regression rate than KD-CAA cases (26.7% vs 66.7%, *p* = 0.004), and the probability of CAA regression in non-KD patients was 0.341 of that in KD patients (*p* = 0.006, OR = 0.341, 95% CI: 0.179–0.647).

*Conclusions*: Various etiologies for Non-KD CAAs are identified. Patients with Non-KD CAAs were observed to have lower inflammatory indexes but poorer recovery than patients with KD CAAs. Therapeutic strategies different than those for KD may be needed for non-KD CAAs.

**Table:** 

**What is Known:** • *Coronary artery aneurysm (CAA) in children is most commonly induced by Kawasaki disease (KD CAA), with a 50 ~ 70% regression rate in 1 to 2 years.* • *CAA induced by diseases other than KD (non-KD CAA) in children is rare and its prognosis remains largely unknown*.
**What is New:** • *Most non-KD CAA cases are caused by coronary artery structural malformations*. • *Non-KD CAA in children has poorer prognosis and lower regression rate compared with KD CAA*. • *In addition to guideline directed anti-platelet and anti-coagulant therapies, treatments targeting the causal factor are necessary for non-KD CAA*.

**What is Known:**

• *Coronary artery aneurysm (CAA) in children is most commonly induced by Kawasaki disease (KD CAA), with a 50 ~ 70% regression rate in 1 to 2 years.*

• *CAA induced by diseases other than KD (non-KD CAA) in children is rare and its prognosis remains largely unknown*.

**What is New:**

• *Most non-KD CAA cases are caused by coronary artery structural malformations*.

• *Non-KD CAA in children has poorer prognosis and lower regression rate compared with KD CAA*.

• *In addition to guideline directed anti-platelet and anti-coagulant therapies, treatments targeting the causal factor are necessary for non-KD CAA*.

## Introduction

Coronary artery aneurysms (CAAs) commonly seen in children with Kawasaki disease (KD) can also be induced by other conditions, including immune vasculitis, infectious diseases, and congenital coronary artery disease [1–5]. Non-KD induced CAAs (non-KD CAAs) are associated with high risks of adverse cardiovascular events, including a high thrombus burden, acute coronary syndrome, and exercise-induced myocardial ischemia with no typical clinical symptoms [3, 6]. The incidence of non-KD CAAs in children is unknown and their clinical features and prognosis are poorly documented. In this case series study, we aimed to investigate pediatric non-KD CAAs to improve the understanding of theses conditions and promote the standardization of the diagnosis and treatment. Additionally, we sought to compare the characteristics of non-KD CAAs and KD CAAs.

## Methods

### Study subjects and data collection

Patients who were admitted to our department and diagnosed with CAA induced by non-KD or KD from January 2022 to December 2023 were included in this study. Medical records were retrieved and demographic characteristics, biochemical parameters, coronary artery involvement and outcomes were retrospectively analyzed.

### Diagnostic criteria

KD was diagnosed according to the criteria established in the 2017 American Heart Association (AHA) Scientific Statement [7]. CAAs were classified by the Z-value method recommended by the AHA as follows: a small CAA has a Z ≥ 2.5 but < 5, a medium CAA has a Z ≥ 5 but < 10, and a giant CAA has a Z ≥ 10 (7). Inclusion criteria are: (1) patients who received no treatment prior to admission; (2) KD patients who were treated at our department with standard IVIG combined with aspirin; (3) patients with CAAs who were given single anti-platelet therapy for small CAAs, dual anti-platelet therapy for medium CAAs, and dual anti-platelet therapy combined with warfarin or low molecular weight heparin (LMWH) for giant CAAs or the risk of thrombosis, in accordance with the 2017 AHA Scientific Statement; and (4) patients who had follow-ups for at least 1 month. Exclusion criteria are: (1) patients who had been treated prior to admission; (2) patients who did not receive regular treatment; and 3) patients who did not return for follow-ups.

### Definition of CAA regression

CAAs in both groups were measured at the time of diagnosis and again at follow-ups at 3 months, 6 months, and 12 months post diagnosis. Complete regression is defined if the Z score < 2.0 and regression to small CAAs is defined if the Z score ≥ 2.5 but < 5.0.

### Statistical analysis

Data normality was determined by the Shapiro–Wilk test. Parametric continuous data were expressed as mean ± standard deviation (SD) and analyzed by two tailed Student’s *t*-test. Nonparametric data were presented as median (Q1, Q3) and analyzed by the Mann–Whitney test. *P* < 0.05 was considered statistically significant. All statistical analyses were performed using the SPSS 23.0 software (IBM Corporation, Armonk, USA) and GraphPad Prism 8.0.2.

## Results

### The etiologies and prognosis of non-KD CAAs

A total of 15 children, 10 males and 5 females aged from 1 month to 13 years were diagnosed with non-KD CAAs. The causes included: unknown etiologies (2 cases), coronary artery fistula (3), Takayasu arteritis (2), virus infection (2), right coronary artery originating from the left coronary sinus (1), restricted cardiomyopathy (1), hypertrophic cardiomyopathy (1), aplastic anemia with agranulocytosis (1), ANCA-associated vasculitis (1), and mucopolysaccharidosis (1) (Table 1). In all non-KD CAA cases, KD was ruled out. Two patients with unknown etiology were found with CAAs accidentally during echocardiography examination; they did not have any manifestations of KD, such as long-time fever, rashes, red eyes, lymphadenopathy, strawberry tongue or peels of extremities. ANCA vasculitis in a patient was P ANCA positive without mucocutaneous symptoms. A patient with parainfluenza viral infection had a fever course less than 5 days without mucocutaneous symptoms or high inflammatory indexes. A patient with EB virus infection had liver and spleen enlargement but no mucocutaneous symptoms. A patient with restrictive cardiomyopathy was diagnosed as idiopathic cardiomyopathy without infiltrative cardiomyopathies or storage diseases.

**Table 1:** Etiologies and prognosis of 15 non-KD induced CAA cases

Number	Gender	Age	Etiology	Involved coronary artery and type	Follow-up	Prognosis
1	Male	1 m	Coronary artery-coronafy sinus fistula complicated with cardiac enlargement	LMCA and LCX with medium CAA	2 days	Died of heart failure
2	Male	8 y	ANCA associated vasculitis	LMCA with small CAA	6 months	Complete regression
3	Female	6 y	Takayasu arteritis	LMCA with small CAA	1 year	Complete regression
4	Female	6 m	Takayasu arteritis	RCA with giant CAA and LMCA with medium CAA	1 year	Regression to small CAA in RCA and complete regression in LMCA
5	Female	3 y	Parainfuenza virus infection	LMCA with small CAA	3 months	Complete regression
6	Female	6 y	Aplastic anemia with agranulocytosis	LMCA with small CAA	2 years	Persistent small CAA
7	Male	10 y	Unknown etiology	LMCA with small CAA	6 months	Persistent small CAA
8	Female	2 y	Mucopolysaccharidosis type I	LMCA with small CAA	3 months	Complete regression
9	Male	12 y	Restrictive cardiomyopathy	LMCA and RCA with small CAA	1 months	Persistent small CAA
10	Male	1 y	Coronary artery—right atrium fistula	LMCA with small CAA	6 months	Persistent small CAA
11	Male	4 y	Coronary artery—right ventricle fistula	LMCA with medium CAA LAD with small CAA	3 months	Persistent medium CAA of LMCA and small CAA of LAD
12	Male	13 y	Chronic active Epstein-Barr virus infection	LMCA with small CAA	1 month	Persistent small CAA
13	Male	7 y	Anomalous origin of the right coronary artery from left coronary sinus	RCA with giant CAA	1 month	Persistent giant CAA with thrombi in RCA dissolved after anticoagulant and dual anti-platelet therapy
14	Male	4 m	Hypertrophic cardiomyopathy	RCA with medium CAA	1 year	Persistent medium CAA
15	Male	7 y	Unknown etiology	LAD with giant CAA	3 months	Persistent giant CAA

*KD* kawasaki disease, *CAA* coronary artery aneurysm, *LMCA* left main coronary artery, *LAD* left anterior descending coronary artery, *LCX* left circumflex artery

A total of 19 CAAs with 3 being giant, 5 medium, and 11 small were found in non-KD patients (Table 1), One patient with a fistula between the right coronary artery and the coronay sinus complicated with cardiac enlargement died of heart failure, despite being treated with positive inotropic agents, diuretics, and vasodilators. One child with the right coronary artery originating from the left coronary sinus had coronary thrombosis that was resolved 2 weeks after anticoagulant therapy. Three patients had complete CAA regression after anti-inflammation therapy, including one with parainfluenza virus infection, one with ANCA-related vasculitis, and one with Takayasu aortitis. One patient with mucopolysaccharidosis also had complete CAA regression 3 months after hematopoietic stem cell transplantation (Table 1).

#### Comparison of non-KD CAAs and KD CAAs

**Fig. 1:**
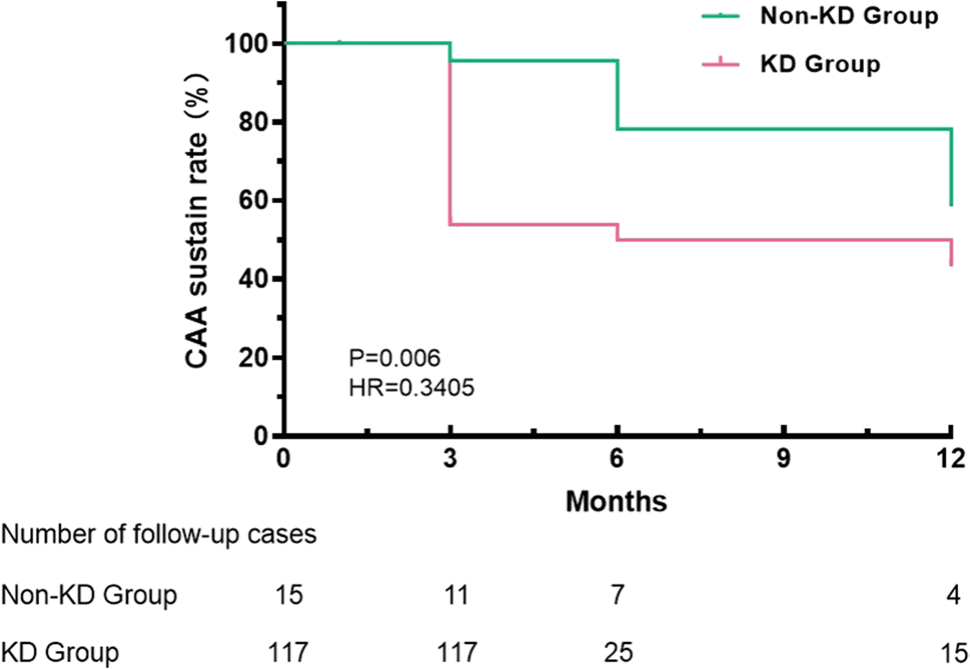
The rate of sustained CAAs in KD and non-KD patients. *CAA* coronary artery aneurysm, *KD* kawasaki disease

As one of the biggest pediatric cardiovascular centers in China, we accept patients from all over the country and treated approximately 750 children with KD during the research period. Of these patients, 117 had CAAs and were included in this study. Compared with KD patients, non-KD patients is older. Blood testing at the time of diagnosis revealed that non-KD patients had higher levels of serum albumin and cardiac troponin T (cTNT), but lower white blood cell (WBC) counts, C reactive protein (CRP), erythrocyte sedimentation rate (ESR) and interleukin 2 receptor (IL-2R) (*p* < 0.05) (Table 2). Giant CAA occurred more frequently in non-KD patients (20%) than in children with KD (6%), although the difference was not statistically significant (*p* = 0.088). In addition, children with Non-KD CAAs had a significantly lower regression rate than KD-CAA cases (26.7% vs 66.7%, *p* = 0.004) (Fig. 1 and Table 2), and the probability of CAA regression in non-KD patients was 0.341 of that in KD patients (*p* = 0.006, OR = 0.341, 95% CI: 0.179–0.647).

**Table 2:** Comparison of demographic data, biochemical parameters, CAA classifications and outcomes between the two groups of patients

Variables	KD group *N* = 117	Non-KD group *N* = 15	*P* values
Age (y)	2.0 [1.0, 3.0]	6.0 [1.5, 7.5]	0.022
Gender (male), n (%)	80 (68.4)	10 (66.7)	1.000
Height (cm)	90.0 [78.0, 104.0]	117.0 [97.0, 132.5]	0.006
Weight (Kg)	12.5 [9.8, 16.3]	17.5 [11.8, 25.3]	0.051
Serum albumin (g/L)	36.50 ± 4.78	40.61 ± 6.84	0.003
WBC (10^9/L)	14.71 [11.89, 18.45]	8.04 [4.74, 11.97]	< 0.001
HGB (g/L)	111.0 [103.0, 117.0]	107.0 [97.0, 138.0]	0.994
PLT (10^9/L)	369.0 [274.0, 481.0]	312.0 [213.0, 464.5]	0.233
CRP (mg/L)	57.66 [32.46, 120.62]	2.49 [0.78, 10.80]	< 0.001
ESR (mm/h)	63.83 ± 30.47	30.17 ± 30.16	0.003
cTnT (ng/mL)	5.10 [3.20, 7.00]	11.60 [4.00, 35.45]	0.03
NT-Pro-BNP (pg/mL)	490.5 [134.6, 1,724.0]	1,593.0[75.2, 3,540.5]	0.372
TNF-α (pg/mL)	18.20 [13.10, 25.90]	16.90 [10.96, 28.40]	0.688
IL-6 (pg/mL)	21.90 [6.57, 53.10]	16.30 [6.00, 35.30]	0.598
IL-10 (pg/mL)	9.11 [5.00, 28.90]	5.00 [5.00, 10.10]	0.14
IL-1β (pg/mL)	5.03 [5.00, 13.20]	5.00 [5.00, 6.32]	0.067
IL-2R (pg/mL)	1,851.00 [1,234.00, 3,090.00]	839.00 [786.00, 1,581.00]	0.012
Follow up (month)	3.00 [3.00, 3.00]	3.00 [2.00, 9.00]	0.801
Giant CAA, n ( %)	7 ( 6.0)	3 (20.0)	0.088
CAA classification			
Small CAA, n (%)	82 (70.1)	9 (60.0)	0.159
Medium CAA, n (%)	28 (23.9)	3 (20.0)	
Giant CAA, n (%)	7 ( 6.0)	3 (20.0)	
CAA regression, n ( %)	78 (66.7)	4 (26.7)	0.004
Death, n ( %)	0 ( 0)	1 (6.7)	0.114
Thrombosis, n (%)	5 (4.3)	1 (6.7)	0.522

Data are expressed as mean ± standard deviation or median [first quartile, third quartile]. *CAA* coronary artery aneurysm, *KD* kawasaki disease, *WBC* white blood cell, *HGB* hemoglobin, *PLT* platelet, *CRP* C reactive protein, *ESR* erythrocyte sedimentation rate, *cTNT* cardiac Troponin T, *NT-Pro-BNP* N terminal-pro-B type natriuretic peptide, *TNF* tumor necrosis factor, *IL* interleukin

## Discussion

Commonly occurring with KD, CAAs induced by other diseases in children are poorly described. In the present study, we investigated 15 non-KD CAA cases and reported the following findings: (1) the etiologies were various for non-KD CAAs, including coronary structural malformations, Takayasu arteritis, infectious diseases, cardiomyopathy, and mucopolysaccharidosis; (2) compared with patients with KD CAAs, children who had non-KD CAAs were older with lower inflammatory indexes and higher levels of cTNT; and (3) non-KD CAAs had a lower regression rate and a higher rate of giant CAA.

The overall incidence of CAA in adults has been reported to range from 0.3% to 5.3% [8, 9], however, the exact incidence of CAA in the pediatric population remains unknown. Apart from KD, other etiologies of CAA have been described, including atherosclerosis, coronary artery structural malformations, Takayasu arteritis, systemic connective tissue diseases (e.g., Marfan syndrome and Ehler-Danlos syndrome), and infections (e.g., EB virus, HIV virus, fungal embolus, syphilis, and Lyme disease) [8–16], some of which were also observed in our patients. The most commonly seen cause for CAAs in our patients is the coronary artery structural malformation, with 3 cases of coronary artery fistula and 1 case of right coronary artery originating from left coronary sinus. A few case reports have shown CAAs with coronary artery fistulas in adults. Lai reported a case of coronary artery fistula complicated with giant CAA and coronary sinus tumor in a 28-year patient [12]. In another case report, Zhaoping described a giant CAA secondary to a coronary right atrium fistula in a 29-year female [10]. It is suggested that CAAs may result from compensatory coronary dilation secondary to distal coronary artery steal, which may be a causal factor related to CAAs caused by coronary artery fistulas. Anomalous origin of the coronary artery is a rare congenital coronary artery malformation, with an incidence of 0.3–1% and a high rate of sudden cardiac arrest [15]. We observed a giant CAA in a child who had the right coronary artery originating from left coronary sinus. Such an phenomenon was also reported in adults. Antelo et al. described a giant CAA in a 39-year-old patient with the left coronary artery originating from the pulmonary artery [17]. In the present study, we also found 2 cases of cardiomyopathy: one with restrictive cardiomyopathy and the other with hypertrophic cardiomyopathy. CAAs secondary to cardiomyopathy have not been documented in the literature. Both restrictive and hypertrophic cardiomyopathy can lead to diastolic dysfunction, which shortens the diastolic period and causes the blood flow to decrease in the coronary artery, resulting in chronic coronary artery compensatory dilation or the formation of CAA. In our case series, one child had a genetic disorder, i.e., mucopolysaccharidosis type I disease. A genetic defect, namely the deficiency of the adenosine deaminase 2 gene, was reported in a 15-month old child with KD CAA [3]. Other genetic diseases, including familial retinal arterial macroaneurysms, polycystic kidney, and Marfan syndrome, have been revealed to be associated with CAAs [13, 18, 19]. Nevertheless, how genetic factors contribute to CAA pathogenesis is unknown and warrants further study.

Although the pathogenesis of CAA remains to be fully elucidated, typical pathological changes in affected coronary arteries have been described, which include the destruction of the tunica media and the degradation of elastic fibers [6–9]. CAA pathological manifestations may vary with different etiologies. Indeed, in the present study, we found that giant CAAs occurred more frequently in patients with non-KD than in patients with KD.

Compared with children with KD CAAs, children with non-KD CAAs had lower levels in inflammatory indexes, reflecting the fact that most of non-KD CAA cases were not associated with inflammation. On the other hand, poorer prognosis and higher rates of cardiac events were observed in non-KD cases, suggesting therapeutic strategies different than those for KD CAAs should be adopted to treat non-KD CAAs.

CAA management is challenging, especially in children, due to limited evidence-based large scale studies. Therefore, early diagnosis and prompt treatment to prevent cardiac events are necessary. Our findings may help clinicians in the diagnosis and intervention of CAAs induced by diseases other than KD.

## Limitations

This study has the following limitations: 1) it is a single center study with a small sample size; 2) it is a retrospective study; and 3) patients had a relatively short follow-up period (median 3 months), which may miss some cases who had CAA regression, although most CAA regression occurred at 3 months after onset according to our own clinical experience.

## Conclusions

Various etiologies of Non-KD CAAs are identified, including coronary structural abnormalities, infectious diseases, cardiomyopathy, and genetic disorders. Patients with non-KD CAAs were observed to have lower inflammatory indexes and poorer outcomes than patients with KD CAAs. Therapeutic strategies different than those for KD may be needed for non-KD CAAs.

## Data Availability

No datasets were generated or analysed during the current study.

## Abbreviations

CAA: Coronary artery aneurysm
CRP: C reactive protein
cTNT: Cardiac troponin T
ESR: Erythrocyte sedimentation rate
IVIG: Intravenous immunoglobulin
KD: Kawasaki disease
NT-Pro-BNP: N-terminal-Pro-B type natriuretic peptide

## References

[1] Libertini R, Wallbridge D, Jones HR, Gunning M, Satur CMR (2018) Giant circumflex artery aneurysm with a coronary sinus fistula. Ann Thorac Surg 106(5):e223–e225 29763599 10.1016/j.athoracsur.2018.04.038

[2] Vasudevan AK, Kumar GA, Rajesh S, Ahamed MZ (2021) IgG4-related coronary aneurysm in a child. Indian J Pediatr 88(6):593 33864603 10.1007/s12098-021-03743-3

[3] Shivpuri A, Sharma R, Mittal J (2023) A Young Child with Fever, Thrombocytosis, and Coronary Aneurysm-Not Kawasaki Disease? Indian J Pediatr 90(1):99 36434492 10.1007/s12098-022-04410-x

[4] Kuo HC (2017) Preventing coronary artery lesions in Kawasaki disease. Biomed J 40(3):141–146 28651735 10.1016/j.bj.2017.04.002 PMC6136281

[5] Cobilinschi CO, Gradinaru E, Saulescu I, Carstea N, Caraiola S, Balanescu AR, Opris-Belinski D (2023) Refractory takayasu’s arteritis with severe coronary involvement-case report and literature review. J Clin Med 12(13):4394 37445428 10.3390/jcm12134394 PMC10342903

[6] Esposito L, Di Maio M, Silverio A, Cancro FP, Bellino M, Attisano T, Tarantino FF, Esposito G, Vecchione C, Galasso G et al (2021) Treatment and outcome of patients with coronary artery ectasia: current evidence and novel opportunities for an old dilemma. Front Cardiovasc Med 8:805727 35187112 10.3389/fcvm.2021.805727 PMC8854288

[7] McCrindle BW, Rowley AH, Newburger JW, Burns JC, Bolger AF, Gewitz M, Baker AL, Jackson MA, Takahashi M, Shah PB et al (2017) Diagnosis, treatment, and long-term management of kawasaki disease: a scientific statement for health professionals from the american heart association. Circulation 135(17):e927–e999 28356445 10.1161/CIR.0000000000000484

[8] Abou Sherif S, Ozden Tok O, Taskoylu O, Goktekin O, Kilic ID (2017) Coronary artery aneurysms: a review of the epidemiology, pathophysiology, diagnosis, and treatment. Front Cardiovasc Med 4:24 28529940 10.3389/fcvm.2017.00024 PMC5418231

[9] Kawsara A, Nunez Gil IJ, Alqahtani F, Moreland J, Rihal CS, Alkhouli M (2018) Management of coronary artery aneurysms. JACC Cardiovasc Interv 11(13):1211–1223 29976357 10.1016/j.jcin.2018.02.041

[10] Zhaoping C, Ximing W, Bin Z, Yanhua D, Juan F, Mei Z (2015) Giant coronary aneurysm secondary to coronary-atrial fistula. J Am Coll Cardiol 65(5):e3 25660938 10.1016/j.jacc.2013.09.088

[11] Yang S, Liang M, Chen G, Yang M, Wu ZK (2020) Giant coronary artery aneurysm combined coronary right ventricle fistula. Circ Cardiovasc Imaging 13(4):e010166 32208735 10.1161/CIRCIMAGING.119.010166

[12] Lai B, Yang Q, Deng M (2024) Coronary artery fistula with giant right coronary artery aneurysm and right coronary sinus tumor. Asian J Surg 47(3):1592–1593 38097496 10.1016/j.asjsur.2023.12.025

[13] Mariucci E, Bonori L, Lovato L, Graziano C, Ciuca C, Pacini D, Di Marco L, Angeli E, Careddu L, Gargiulo G et al (2021) Coronary artery aneurysms in patients with marfan syndrome: frequent, progressive, and relevant. Can J Cardiol 37(8):1225–1231 33711475 10.1016/j.cjca.2021.03.002

[14] Arboine L, Palacios JM (2018) Left main coronary artery aneurysm. N Engl J Med 378(23):e32 29874527 10.1056/NEJMicm1708877

[15] Nagashima K, Hiro T, Fukamachi D, Okumura Y, Watanabe I, Hirayama A, Tanaka M, Tanaka T, Takamisawa I, Taguchi I et al (2020) Anomalous origin of the coronary artery coursing between the great vessels presenting with a cardiovascular event (J-CONOMALY Registry). Eur Heart J Cardiovasc Imaging 21(2):222–230 31185091 10.1093/ehjci/jez076

[16] Cho S, Jeon KN, Bae K (2015) Anomalous origin and aneurysm of the right coronary artery associated with congenital bicuspid aortic valve: MDCT findings. Springerplus 4:426 26290805 10.1186/s40064-015-1214-1 PMC4539311

[17] Antelo M, Freire D, Dendi A, Parma G, Fernandez N, Picarelli D (2023) Anomalous origin of the left coronary artery from the pulmonary artery associated with right coronary giant aneurysm. World J Pediatr Congenit Heart Surg 14(2):238–240 36464765 10.1177/21501351221135767

[18] Shah MA, Alqahtani A, Alshahrani ST, Alshehri HZ (2022) Giant coronary artery aneurysm associated with familial retinal artery macroaneurysm: a case report. Eur Heart J Case Rep 6(2):ytac057 10.1093/ehjcr/ytac057 PMC8922714 35299703

[19] Neves JB, Rodrigues FB, Lopes JA (2016) Autosomal dominant polycystic kidney disease and coronary artery dissection or aneurysm: a systematic review. Ren Fail 38(4):493–502 26888492 10.3109/0886022X.2016.1144209

